# Proteomic analysis of human follicular fluid from fertile women

**DOI:** 10.1186/s12014-015-9077-6

**Published:** 2015-03-03

**Authors:** Alberuni M Zamah, Maria E Hassis, Matthew E Albertolle, Katherine E Williams

**Affiliations:** Department of Obstetrics and Gynecology, Division of Reproductive Endocrinology and Infertility, University of Illinois at Chicago College of Medicine, Chicago, IL 60612 USA; Sandler-Moore Mass Spectrometry Core Facility, University of California at San Francisco, San Francisco, CA 94143 USA; Center for Reproductive Sciences and the Department of Obstetrics and Gynecology, University of California at San Francisco, San Francisco, CA 94143 USA

**Keywords:** Follicle development, Follicular fluid, Human, Proteomics

## Abstract

**Background:**

Follicular fluid is a unique biological fluid in which the critical events of oocyte and follicular maturation and somatic cell-germ cell communication occur. Because of the intimate proximity of follicular fluid to the maturing oocyte, this fluid provides a unique window into the processes occurring during follicular maturation. A thorough identification of the specific components within follicular fluid may provide a better understanding of intrafollicular signaling, as well as reveal potential biomarkers of oocyte health for women undergoing assisted reproductive treatment. In this study, we used high and low pH HPLC peptide separations followed by mass spectrometry to perform a comprehensive proteomic analysis of human follicular fluid from healthy ovum donors. Next, using samples from a second set of patients, an isobaric mass tagging strategy for quantitative analysis was used to identify proteins with altered abundances after hCG treatment.

**Results:**

A total of 742 follicular fluid proteins were identified in healthy ovum donors, including 413 that have not been previously reported. The proteins belong to diverse functional groups including insulin growth factor and insulin growth factor binding protein families, growth factor and related proteins, receptor signaling, defense/immunity, anti-apoptotic proteins, matrix metalloprotease related proteins, and complement activity. In a quantitative analysis, follicular fluid samples from age-matched women undergoing in vitro fertilization oocyte retrieval were compared and 17 follicular fluid proteins were found at significantly altered levels (p < 0.05) between pre-hCG and post-hCG samples. These proteins belong to a variety of functional processes, including protease inhibition, inflammation, and cell adhesion.

**Conclusions:**

This database of FF proteins significantly extends the known protein components present during the peri-ovulatory period and provides a useful basis for future studies comparing follicular fluid proteomes in various fertility, disease, and environmental exposure conditions. We identified 17 differentially expressed proteins after hCG treatment and together these data showed the feasibility for defining biomarkers that illuminate how the ovarian follicle microenvironment is altered in various infertility-related conditions.

**Electronic supplementary material:**

The online version of this article (doi:10.1186/s12014-015-9077-6) contains supplementary material, which is available to authorized users.

## Introduction

The biologic niche where oocyte growth and maturation occurs within the ovary is termed the ovarian follicle. The maturing ovarian follicle is separated from other structures within the ovary by a basement membrane and has both somatic cell components (mural and cumulus granulosa cells) as well as the germ cell component (oocyte). During follicular development there is a coordination of development between the somatic cells and the oocyte. This regulation and coordination involves endocrine, as well as paracrine and autocrine signaling within the specialized microenvironment of the human ovarian follicle. As a follicle is undergoing maturation through the secondary to antral stages, it develops a fluid filled cavity termed the antrum. The antrum of the developing follicle is filled with fluid (termed follicular fluid) which is a select ultrafiltrate of plasma that has been modified by secretion and uptake of specific components by the cells within the follicle itself [1,2]. The ability of proteins to cross this blood-follicle barrier is based both on molecular weight as well as charge characteristics [3]. Follicular fluid is the medium by which signaling mediators are transported in and out of the follicle, as well as within the follicle between various cell types. Given that intrafollicular communication is critical for normal oocyte development and reproduction, much effort has been directed at better understanding intrafollicular signaling. It is evident that there is communication from the mural granulosa cells to the cumulus complex [4-6]; from the cumulus complex to the oocyte; and from the oocyte back to the somatic compartment [7-10]. These signaling events can be mediated by soluble small molecules via gap junctions or lipid signals (e.g., cGMP, ffMAS, sphingosine-1-p) [11-14], but the majority of the components for paracrine intrafollicular signaling identified to date involve peptide hormones [15-19].

Because of the intimate proximity of follicular fluid to the maturing oocyte, this biologic fluid provides a unique window into the processes occurring during follicular maturation. The specific components within follicular fluid will help us better understand intrafollicular signaling, as well as reveal potential biomarkers of oocyte health for women undergoing assisted reproductive ART treatment. In this study we used a mass spectrometry approach to identify the proteins present in human follicular fluid (FF) to better understand the paracrine signals at play in the peri-ovulatory time period. Prior mass spectrometry studies have been performed on FF samples obtained from IVF patients. Studies have evaluated fluid from presumably healthy women (such those with male factor infertility) [20,21]; from women who have had successful IVF compared to those who did not [22]; in mature versus immature follicles [23]; and in pathologic states such as infertility [24-26] or in women suffering from repetitive pregnancy loss [27]. In order to develop a better understanding of this matrix, we set out to explore the proteome of healthy young fertile women to better define the normal repertoire of the intra-follicular environment during oocyte maturation. In this study we assayed FF from anonymous oocyte donors undergoing IVF oocyte retrieval, with the hypothesis that the proteins identified will allow for a better understanding the follicular fluid milieu in normal healthy reproductive age women. Human chorionic gonadotropin (hCG) is routinely used as a single injection to induce the final stage of follicle and oocyte maturation during IVF treatment. Therefore, we have also performed a comparison of the follicular fluid proteome from pre-hCG and post-hCG follicular fluid samples to reveal which proteins are significantly changed during follicular maturation.

## Results and discussion

**Figure 1:**
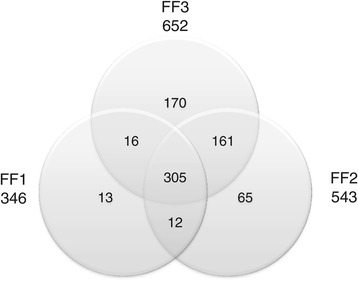
**Overlap of proteins identified at 5% false discovery rate in three follicular fluid samples from ovum donors undergoing ovarian stimulation for assisted reproductive technology.** A total of 742 proteins were identified, of which 305 were found in all three samples. Source accessions for the identified proteins are listed in Additional file 1: Table S1.

Proteomic analysis of biological fluids is complicated by the large dynamic range of protein concentrations, spanning ten orders of magnitude, greatly exceeding that of any methods used for proteomic analysis [28]. Follicular fluid is a plasma filtrate with the concomitant large dynamic range of protein concentrations that make detection of lower abundance proteins, where new clinically useful marker proteins might be found, challenging. To maximize the depth of coverage, individual follicular fluid samples from 3 ovum donors were immunodepleted of the 14 most abundant plasma proteins and extensively fractionated using alkaline pH reverse phase chromatography of peptides prior to LC-MS/MS analysis. A total of 742 distinct follicular fluid proteins were detected, with 305 of these found in all three samples (Figure 1). A list of the follicular fluid proteins detected in each sample, with score, Swiss-Prot accession numbers, and gene names is presented in Additional file 1: Table S1.

Several groups have previously analyzed post-hCG FF by using mass spectrometry-based methods [9,21-26,29,30]. Twigt et al. identified 246 proteins from FF by using SDS-PAGE and isoelectric focusing (IEF) fractionation prior to LC-MS/MS [31]. A comparison of the combined list of proteins obtained using both fractionation methods, shows that the majority of proteins, 189/246 were also detected in this study (Additional file 1: Table S1). Using immunodepletion, SDS-PAGE, IEF, and strong cation exchange separation strategies, Ambekar et al. recently reported 480 FF proteins [21]. The FF proteome described here includes 297 of those proteins (Additional file 1: Table S1). We have extended the follicular fluid proteome with an additional 413 high confidence distinct proteins. We detected FF proteins not previously reported using MS methods, e.g., cell migration-related MEMO1, S100-A7, and secretogranin-1 and −2*.*

Plasma proteins contribute to the composition of all body fluids, e.g., peritoneal fluid, urine, synovial fluid, saliva, and cerebrospinal fluid. (reviewed in [32,33]) The constituents and concentrations of plasma-derived proteins in body fluids are dependent on the molecular weight, charge, solubility, microvascular permeability and the molecular structure of the compartment. A comparison of the FF proteome to the list of 1929 high confidence human plasma proteins combined from 91 experiments [34] showed 585 proteins in common (Additional file 1: Table S1), a not unexpected finding given the plasma-filtrate origin of the follicular fluid. The coagulation factors are potentially correlated to the inflammatory-related peptides present in FF and have relevant roles within the follicle [35,36]. Thrombin, which found in lower levels in FF as compared to serum [37], was recently shown to be an intra-ovarian signal for optimal follicular luteinization in mice [38]. Antithrombin was found in decreased levels in FF from IVF patients with successful outcomes [22]. To investigate the differences in the FF proteome, which is compartmentalized by a basement membrane to that of an extracellular fluid where there is no barrier, we compared the proteins to a recently published synovial fluid (SF) proteome [39]. Of the 575 SF proteins reported, 321 (56%) were also detected in FF and the overwhelming majority of these (308) were detected in plasma (Additional file 1: Table S1).

**Figure 2:**
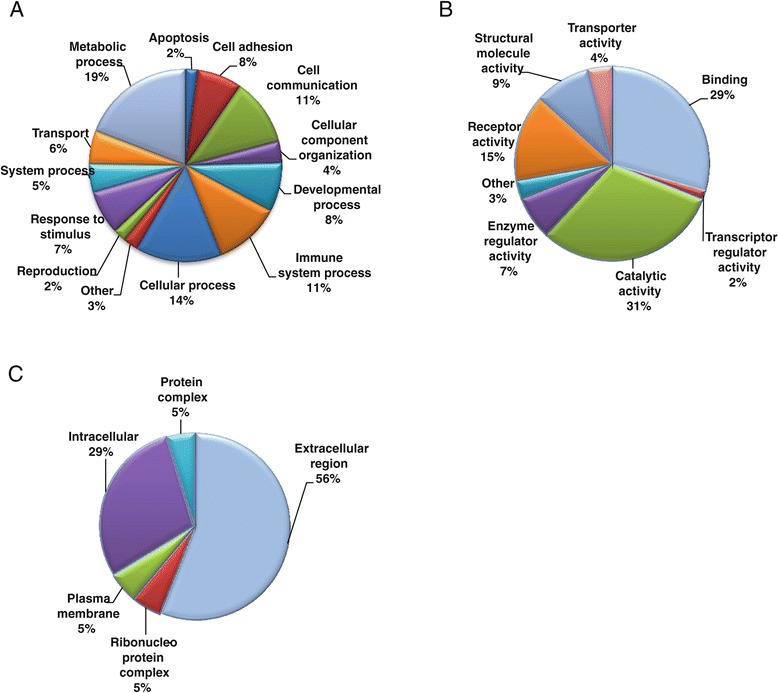
**Gene ontology analysis of the proteins identified in human follicular fluid.** Proteins were classified according to **A)** biological processes, **B)** molecular function, and **C)** cellular compartment. Results are displayed as percent of genes classified to a category over the total number of class hits.

The FF proteome was analyzed to determine gene ontology annotations for biological processes, molecular functions, and cellular compartment. The majority of the follicular fluid proteins detected are involved in metabolic processes (19%), cellular processes (14%), cellular communication (11%), and immune responses (11%) (Figure 2A). A significant number are involved in response to stimulus and developmental processes. The top molecular function categories (Figure 2B) were catalytic activity (31%), binding (29%), and receptor activity (15%). Classification based on the subcellular localization (Figure 2C) indicated that 56% of proteins were extracellular.

Multiple proteins associated with the inflammatory response were detected: the complement proteins, interleukin-related peptides, proteins regulating chemoattraction (attractin, macrophage migration inhibitory factor, collectin-11), and serine proteases (serine protease-1, 2, and 23). Activation of the inflammatory cascade is required for normal ovulation events, and inhibitors of inflammation such as COX-2 inhibitors have been shown to disrupt ovulation [40,41]. Therefore it is not surprising that several of the proteins in follicular fluid during the peri-ovulatory time are related to inflammation [42]. The super family of serine protease inhibitors (serpins) are involved in coagulation, fibrinolysis, and inflammation [43]. Prior studies, primarily using ELISA methods, have described numerous cytokines and chemokines present in human follicular fluid form patients undergoing IVF, although their relationship to IVF success is debatable [44,45]. In a recent publication, Bianchi et al. [46], using 2-DE and LC-MS for proteomic analysis of follicular fluid from women undergoing IVF, reported inflammation being the predominant class of proteins identified.

A complex program of signaling events is required for follicular maturation [47,48]. Multiple FF proteins with roles in signaling were identified, including insulin growth factor (IGF) and IGF binding proteins, growth factor or growth factor related proteins, anti-apoptotic proteins, and matrix metalloprotease related proteins (Table 1). The intra-ovarian IGF system has been extensively studied in terms of its effects on folliculogenesis and steroidogeneisis (reviewed in [49]). Matrix metalloproteases (MMPs) play a role in the processing of hormones to their active forms, as well as in a variety of other processes including extracellular matrix remodeling and inflammation. MMP-9 has been suggested to be a correlated with IVF success [50]. TIMP1 has been detected in human luteinized granulosa cells, as well as in the ovarian cells from other species [51-53]. Apoptosis of cumulus cells in the oocytes has recently been associated with competence [54].

**Table 1:** **Follicular fluid proteins with functional roles in signaling**

**Accession**	**Gene Name**	**Protein Name**
**IGF related**		
P35858	IGFALS	Insulin-like growth factor-binding protein complex acid labile subunit
P05019	IGF1	Insulin-like growth factor I
P01344	IGF2	Insulin-like growth factor II
P08833	IGFBP1	Insulin-like growth factor-binding protein 1
P18065	IGFBP2	Insulin-like growth factor-binding protein 2
P17936	IGFBP3	Insulin-like growth factor-binding protein 3
P22692	IGFBP4	Insulin-like growth factor-binding protein 4
P24593	IGFBP5	Insulin-like growth factor-binding protein 5
P24592	IGFBP6	Insulin-like growth factor-binding protein 6
Q16270	IGFBP7	Insulin-like growth factor-binding protein 7
Q13219	PAPPA	Pappalysin-1
**Metalloproteinase related**		
P08253	MMP2	72 kDa type IV collagenase
P14780	MMP9	Matrix metalloproteinase-9
Q9UHI8	ADAMTS1	A disintegrin and metalloproteinase with thrombospondin motifs 1
Q76LX8	ADAMTS13	A disintegrin and metalloproteinase with thrombospondin motifs 13
Q6UY14	ADAMTSL4	ADAMTS-like protein 4
Q96KN2	CNDP1	Beta-Ala-His dipeptidase
P15169	CPN1	Carboxypeptidase N catalytic chain
P01033	TIMP1	Metalloproteinase inhibitor 1
P16035	TIMP2	Metalloproteinase inhibitor 2
O95980	RECK	Reversion-inducing cysteine-rich protein with Kazal motifs
**Anti-apoptotic**		
P99999	CYCS	Cytochrome c
P81605	DCD	Dermcidin
Q9Y4L1	HYOU1	Hypoxia up-regulated protein 1
P02750	LRG1	Leucine-rich alpha-2-glycoprotein
P83110	HTRA3	Probable serine protease HTRA3
P49908	SEPP1	Selenoprotein P
**Other growth factor & related**		
P15514	AREG	Amphiregulin
Q9Y5C1	ANGPTL3	Angiopoietin-related protein 3
P01019	AGT	Angiotensinogen
O94985	CLSTN1	Calsyntenin-1
Q16627	CCL14	C-C motif chemokine 14
P26992	CNTFR	Ciliary neurotrophic factor receptor subunit alpha
Q96HD1	CRELD1	Cysteine-rich with EGF-like domain protein 1
Q9UBP4	DKK3	Dickkopf-related protein 3
Q13822	ENPP2	Ectonucleotide pyrophosphatase/phosphodiesterase family member 2
Q8N441	FGFRL1	Fibroblast growth factor receptor-like 1
Q9Y625	GPC6	Glypican-6
P10912	GHR	Growth hormone receptor
Q04756	HGFAC	Hepatocyte growth factor activator
P08581	MET	Hepatocyte growth factor receptor
P26927	MST1	Hepatocyte growth factor-like protein
P07333	CSF1R	Macrophage colony-stimulating factor 1 receptor
P10721	KIT	Mast/stem cell growth factor receptor
Q7Z7M0	MEGF8	Multiple epidermal growth factor-like domains protein 8
O14786	NRP1	Neuropilin-1
P30086	PEBP1	Phosphatidylethanolamine-binding protein 1
P36955	SERPINF1	Pigment epithelium-derived factor
Q99435	NELL2	Protein kinase C-binding protein NELL2
Q9HCB6	SPON1	Spondin-1
Q03167	TGFBR3	Transforming growth factor beta receptor type 3
Q15582	TGFBI	Transforming growth factor-beta-induced protein ig-h3
P35590	TIE1	Tyrosine-protein kinase receptor Tie-1
P30530	AXL	Tyrosine-protein kinase receptor UFO
P35916	FLT4	Vascular endothelial growth factor receptor 3
Q6EMK4	VASN	Vasorin

In addition to plasma-derived proteins, another source of proteins within the follicular fluid is the somatic cells within the follicle, which make up the bulk of the protein constituent of the follicle. Many of the proteins detected were intracellular and may represent events such as cumulus separation from the wall in the mature peri-ovulatory follicle and proteolysis of the follicle wall in preparation for ovulation could release intracellular components. We note that multiple intracellular protein fragments are routinely identified from human plasma [34,42,55,56] and FF [21,31]. The cumulus matrix undergoes expansion concomitant with considerable extracellular matrix remodeling at the peri-ovulatory time period. This is congruous with the presence of several extracellular matrix components, e.g. (laminin subunits, ECM1, Fibrillin-1, CGAT1) present at significant levels in the follicular fluid. Furthermore, many of these proteins are known to be abundantly secreted by granulosa cells in the periovulatory time as the cumulus cells begin to expand and separate in a hyaluronan matrix in preparation for ovulation and fertilization. The proper expansion of this matrix is critical for normal sperm penetration and fertilization events to occur [57].

The maturing oocyte, although the largest cell in the human body, contributes negligibly to the bulk of the secreted protein in the follicle fluid. For example, two key oocyte secreted factors (OSF), growth differentiation factor 9 (GDF-9) and bone morphogenic protein 15 (BMP-15) [8] were not detected. This inability to detect oocyte-specific secreted proteins using untargeted mass spectrometry methods [20-26,29-31,58], including our own analyses, likely reflects the much lower abundance of oocyte secreted factors in the follicular fluid relative to the other proteins. Targeted proteomics approaches with enhanced the sensitivity have the potential to detect OSFs present at the tens of ng/mL level [59-61].

Numerous ovarian proteins were detected including follistatin, folllistatin-related proteins 3 and 4, inhibin -alpha, −betaA, −betaB, −betaC, oviduct specific glycoprotein, secreted protein acidic and rich in cysteine (SPARC), pappalysin, and out at first protein homolog (OAF). Some of these were detected for the first time at the protein level in human follicular fluid (Additional file 1: Table S1). The calcium-binding SPARC protein is expressed in elevated levels in tissues undergoing remodeling and wound repair. SPARC has been shown to be significantly increased in luteinized granulosa cells and is regulated by pro-angiogenic and extracellular matrix factors during the folliculo-luteal transition [62]. Pappalysin has also been detected in serum, and in a recent analysis was reported at elevated levels in the serum of women suffering from preeclampsia compared to normotensive women [63].

Identification of putative markers at the protein level paves the way for rapid tests of oocyte competence, but sensitivity is key to the success of these approaches. We compared the proteins found in FF with microarray data acquired from floating granulosa (predominantly mural granulosa) and cumulus granulosa cells isolated at the time of oocyte retrieval to determine whether we could detect cellular proteins that are secreted or shed [64]. Communication between the oocyte and the surrounding somatic cells is essential for acquisition of competence (reviewed in [65,66]). A total of 586 FF proteins were found at the transcript level in these cell types (Additional file 1: Table S1). Sixty-three showed increased mRNA expression in the cumulus cells (>1.5 fold), while 55 transcripts showed increased expression in the mural cell transcriptome (>1.5 fold). Thus, proteomic approaches such as this one have sufficient sensitivity to detect protein abundance changes in FF that may arise from the mural and/or cumulus granulosa cell constituents of the follicle.

To quantify differences in protein abundances after hCG treatment, FF proteins from 6 individuals (3 pre-hCG treatment and 3 post-hCG treatment were compared using iTRAQ mass tags to measure relative abundance changes. The ratios from each group were compared using Student’s *t*-test and 17 proteins were found to be significantly different (p < 0.05). Eleven proteins were significantly higher in the post-hCG samples compared to the pre-hCG samples, and six proteins were significantly lower in the post-hCG samples compared to the pre-hCG samples (Table 2). Prior studies have shown that the majority of these proteins have been detected in FF by orthogonal detection methods such as Western blotting or ELISA. For example prostatic acid phosphatase, metalloproteinase inhibitor 1, alpha-2-HS-glycoprotein, gelsolin, prothrombin, coagulation factor X, inhibin alpha, histidine-rich glycoprotein and extracellular matrix protein 1 have been detected in human follicular fluid by orthogonal methods [67-73]. Further, 10 of the 17 proteins had transcripts that were present in both mural and cumulus granulosa cells (Additional file 1: Table S1). Alpha-2-HS-glycoprotein, N-acetylmuramoyl-L-alanine amidase, prothrombin, pantetheinase, complement component C8 alpha chain, Beta-Ala-His dipeptidase and histidine-rich glycoprotein are presumably not of granulosa cell origin based on the transcriptome comparison. This provides important validation of the iTRAQ proteins detected, although a limitation of our study is that the levels of changes could not be validated within our own samples due to sample volume limitations. The proteins showing differences belonged to a variety of functional processes, including protease inhibition, inflammation, angiogenesis, and cell adhesion. Complement C8 alpha chain was found in higher abundance (1.4-fold) in pre-hCG FF. The role of the complement factors in follicle maturation is unclear, though it has been postulated that they are possible oocyte maturation factors [74]. Another complement factor, C3, was differentially expressed in women with severe ovarian hyperstimulation syndrome [69].

**Table 2:** **Follicular fluid proteins with altered abundance after hCG treatment**

**Protein**	**Accession**	**Gene Name**	**p value**	**Relative abundance**
**Increased abundance post-hCG**				
Prostatic acid phosphatase	P15309	PPAP	1.1E-06	22.0
Metalloproteinase inhibitor 1	P01033	TIMP1	8.3E-04	4.4
Alpha-2-HS-glycoprotein	P02765	FETUA	7.5E-03	2.4
N-acetylmuramoyl-L-alanine amidase	Q96PD5	PGRP2	3.1E-03	2.4
Complement component C7	P10643	CO7	1.1E-02	2.2
Gelsolin	P06396	GELS	1.1E-02	2.0
Vitronectin	P04004	VTNC	2.2E-02	1.8
Prothrombin	P00734	THRB	5.0E-03	1.7
Carboxypeptidase N subunit 2	P22792	CPN2	2.6E-02	1.7
Cystatin-C	P01034	CYTC	4.8E-02	1.5
Extracellular matrix protein 1	Q16610	ECM1	3.5E-02	1.5
**Decreased abundance post-hCG**				
Pantetheinase	O95497	VNN1	4.7E-02	1.3
Complement component C8 alpha chain	P07357	CO8A	2.6E-02	1.4
Coagulation factor X	P00742	FA10	4.5E-02	1.9
Beta-Ala-His dipeptidase	Q96KN2	CNDP1	3.0E-02	3.8
Inhibin alpha chain	P05111	INHA	1.2E-02	4.4
Histidine-rich glycoprotein	P04196	HRG	1.9E-02	5.5

TIMP-1 has been shown to be expressed in luteinized granulosa cells of humans [52], and was found in 4.4- fold higher levels in post-hCG samples. MMPs and tissue inhibitor of metalloproteinases (TIMPs) have been reported to have multiple effects on ovarian function, particularly remodeling the extracellular matrix [75]. A study in endometriosis patients undergoing IVF showed that mature follicles yielding MII oocytes had significantly higher TIMP1 levels compared to follicles yielding immature germinal vesicle oocytes, and that embryos of good morphology were correlated with follicles with higher TIMP1 [73]. Exposure of rats to persistently elevated levels of TIMP1, designed to emulate endometriosis, showed an overall negative affect on ovarian function as it disturbed folliculogenesis and lowered the number of corpora lutea (CL) formed [53]. Inhibin alpha was one of the most up-regulated proteins pre-hCG, consistent with prior studies showing significant expression specifically in granulosa cells [76] as well as its known regulation by LH signaling [77,78]. These proteins have the potential to be biomarkers of normal luteinization and, in combination, could be used as a panel to assay follicular health at the folliculo-luteal transition. The secreted protein extracellular matrix protein 1 (ECM1) is a glycoprotein that inhibits the activity of MMP9 [79]. Higher FF and serum MMP9 levels are associated with IVF success [50]. Thus, levels of ECM1 could be a predictor of oocyte quality.

Histidine-rich glycoprotein (HRG) is an abundant plasma protein that binds a variety of ligands including fibrinogen, heparin, and thrombospondin and has roles in angiogenesis, coagulation, and the immune system [80-84]. HRG has been previously reported in FF [71] and was found in our study to be 5.5-fold higher levels in pre-hCG samples. A single nucleotide polymorphism (C633T) in HRG, which results in a serine to proline at position 186, is associated with primary recurrent miscarriage [85] and a lower pregnancy rate in IVF patients [86]. The tryptic fragment encompassing this site is only 3 amino acids in length, thus too short to be detected by the LCMS methods used in this study. The angiogenic factors fibroblast growth factor (FGF) and vascular endothelial growth factor (VEGF) are produced by the pre-ovulatory follicle and may be key regulators of the angiogenic balance in the follicle [87]. Elevated VEGF levels are associated with unfavorable clinical IVF outcomes such as ovarian hyperstimulation syndrome (reviewed in [88]) and dissolved oxygen content of the follicle, which is related to VEGF, has been correlated with poor oocyte quality [89]. HRG negatively regulates VEGF signaling [90], and HRG levels could be a functional link to between VEGF levels [10,77,91] and responses to stimulation and IVF outcome.

One of the most down-regulated proteins pre-hCG was prostatic acid phosphatase. Lower levels of this protein in follicle fluid are associated with immature oocytes (as assessed by fertilization) [70], in accordance with the levels seen in pre-hCG follicles. Furthermore, in addition to lower levels of prostatic acid phosphatase from pre-HCG FF, we also found lower levels of prothrombin and PGRP2. Recently, Severino et al., [92] used a similar MS approach in FF from IVF patients who conceived versus those who did not and found these three proteins were more abundant in successful IVF. This is consistent with our findings, since pre-hCG follicles do not contain oocytes competent for fertilization. Potentially, a panel of proteins that are regulated during hCG administration would yield a protein signature of optimal luteinization for an individual follicle.

This comparative proteomics analysis provided information about abundance changes in follicular phase fluid (pre-hCG) vs. at the luteal transition (post-hCG), to our knowledge the first such analyses using pre-hCG follicular fluid. Our comparative proteomics analysis showed relatively few significant differences in protein expression from pre- versus post-hCG follicular fluid despite the knowledge that several proteins related to intra-follicular signaling are known to either be up-or down-regulated at the time of the LH surge (e.g., EGF-like growth factors, C-type natriuretic peptide [6,93]). The reason for this is that these proteins were below the detection limit of the iTRAQ portion of our study. However, our unbiased mass spectrometry analysis did detect the EGF-like growth factor amphiregulin, which is present at the average level of 30 ng/ml in post-hCG samples [93]. Therefore it is likely that a combination of sample heterogeneity and preferential detection of higher abundance proteins were the reasons for the relatively low number of iTRAQ differences seen. Our analysis was performed on follicle fluid obtained from ovum donors after the granulosa cells were exposed to an artificial luteinization signal from exogenous hCG administration. Given inter-patient variability, it would be of great benefit to be able to compare this to unluteinized follicle fluid from within the same patients (ideally with serum samples as controls) to allow for enhanced detection of pathways specifically activated by the LH surge and to better understand the intrafollicular environment during the earlier stages of follicle development. Of the FF proteins found to be significantly different between pre-hCG and post-hCG samples, some have putative or known functions in follicle maturation. Others may represent biomarkers of follicle maturation, although their functional role, if any, remains uncertain. The proteins described here will provide a framework for potentially assessing the competence of an oocyte from a given follicle.

## Conclusions

This analysis has been the most comprehensive proteomic evaluation of human follicular fluid in a single study to date, with 742 distinct proteins identified. This extends the FF proteome to 982 high confidence proteins, underscoring the utility of multiple, orthogonal protein and peptide methodologies for comprehensive examination of this complex proteome. This database of FF proteins provides a useful basis for studies comparing follicular fluid proteomes in various fertility, disease, and environmental exposure conditions. In particular, alterations in the protein complement of FF from infertile patients with varying etiologies such as advanced female age, endometriosis, or polycystic ovarian syndrome could be compared to our study on fertile egg donors to improve our understanding of how the ovarian follicle microenvironment is altered under these conditions.

The goal of the present study was to characterize the follicular fluid proteome from fertile women and quantify differences in protein abundances after the hCG administration. A comparison with microarray data from mural granulosa cells and cumulus granulosa cells isolated at the time of oocyte retrieval [64] showed that we could detect many differentially expressed transcripts at the protein level in FF. This ability to detect proteins that may be derived from the somatic cell repertoire of the follicle allows for a functional analysis of these distinct compartments during follicular maturation. Differences in the cumulus cell transcriptome, for instance, have been correlated with IVF outcomes [94-96]. To improve our understanding of normal human intrafollicular dynamics, we performed an untargeted quantitative analysis to reveal biomarker candidates and/or signaling cascades active during follicle growth and maturation. The proteins identified here provide an additional layer of understanding of which proteins are expressed during this time and can provide a method of assessing the somatic compartment of the human follicle. Future directions should focus on targeted protein quantitation from individual follicular aspirates as the basis for non-invasive assessment of oocyte quality. The proteins described here that change in response to hCG may be detected in serum or follicular fluid as markers of premature or appropriate luteinization to allow for improved ovarian stimulation outcomes.

## Methods

### Research approval

All subjects gave consent for donation of follicular fluid for research purposes as part of a UCSF Institutional Review Board approved IVF tissue bank protocol.

### Source and collection of human follicular fluid samples

Patients undergoing assisted reproductive technology (ART) by standard ovarian stimulation protocols were recruited to collect follicular fluid (FF). Details regarding the stimulation parameters, oocyte retrieval and FF preparation and storage have been previously described [93,97]. For all experiments, FF was obtained from mature sized follicles (≥16 mm diameter). For the comprehensive proteomics analysis, follicular fluid from three anonymous ovum donors (all age <30) receiving down-regulated ovarian stimulation protocols were used. Each patient had either one or two aspirates collected at the time of oocyte retrieval (36 hours after hCG treatment for all post-hCG samples) and only a single FF sample from each donor was used for mass spectrometry analysis.

For relative quantification, pre-hCG FF was obtained from three patients: i) a single ovum donor who did not inject hCG and ii) two stimulated IVF patients with single lead follicles that were aspirated to allow salvage of ovarian stimulation. For the post-hCG samples, FF from three age-matched donors, using the same type of ovarian stimulation protocol, were used.

### Sample preparation for mass spectrometry

The protein concentrations were determined using BCA Assay (Thermo Scientific, San Jose, CA). Follicular fluid samples were processed according to the manufacturer’s instructions using a MARS-Hu-14 immunodepletion column from Agilent Technologies (Santa Clara, CA). The protein flow-through fraction was collected and desalted by using a 3 kDa molecular weight cutoff (MWCO) centrifugal concentrator (Sartorius AG, Goettingen, Germany). Immunodepleted samples were denatured using 6 M urea, cysteines reduced with 500 mM dithiothreitol (DTT) and alkylated with 500 mM iodoacetamide (IAA) followed by an over-night incubation with sequencing grade trypsin (Promega, Madison, WI) at 37°C. The resulting peptides were acidified with formic acid and desalted using Oasis HLB Extraction Cartridges (Waters Corporation, Milford, MA). For iTRAQ analysis, a 50 μg aliquot immunodepleted follicular fluid sample from 6 patients (3 pre-hCG and 3 post-hCG treatment, age-matched) was digested with trypsin and labeled with 8plex iTRAQ reagent according to manufacturer’s protocol (AB Sciex, Foster City, CA). A pool comprised of equal amounts of protein from each of the post-hCG samples was labeled and used as the reference standard. Both labeled and unlabeled samples were subjected to offline peptide fractionation by using a Paradigm MS4 HPLC System (Michrom, Auburn, CA) equipped with a Zorbax Extend-C18 column (4.6 × 100 mm, 3.5 μm particle size, Agilent Technologies, Santa Clara, CA) and a guard column of the same packing material. A 100 μg sample of protein digest was reconstituted in 100 μL of Solvent A (0.1% NH_4_OH, pH 10). Peptides were eluted using a linear gradient of 2-50% Solvent B (0.1% NH_4_OH in acetonitrile) over 30 min. and 30 fractions were collected. The fractions were vacuum-dried, reconstituted in 0.1% formic acid, and stored at −80°C until analysis.

### LC-MS/MS and bioinformatics

For protein identification, peptides were analyzed by LC-MS/MS using a nanoLC Ultra system (Eksigent Technologies, Dublin, CA) interfaced with a LTQ Orbitrap Velos mass spectrometer (Thermo Scientific, San Jose, CA). Peptides were separated using an Acclaim PepMap100 C18 column (75 μm i.d. × 15 cm, 3 μ, 100 Å) with a linear gradient of 2-40% B (98% ACN, 0.1% FA) over 60 minutes. MS data were acquired using an LTQ Orbitrap Velos with data-dependent ion selection consisting of the initial MS scan (m/z 350–1600) followed by eight MS/MS scans (m/z 100–1600). The .raw files were processed by Mascot Daemon v.2.2.2 (Matrix Science, Boston, MA) to generate mgf files. Data from the iTRAQ labeled samples were acquired using the same LC conditions using a nano2D MDLC (Eksigent) interfaced with a QSTAR Elite mass spectrometer (AB Sciex). The Paragon algorithm in ProteinPilot v.4.2 (AB Sciex, Foster City, CA) was used for protein identification and quantification and false discovery rates (FDRs) assessed by decoy database searching [98]. Data were searched against the SwissProt database v20120222 using the following parameters: carbamidomethylation of cysteines, trypsin enzyme, *Homo sapiens* species filter, and thorough search effort. Proteins detected with 5% local FDR were reported. To determine significant changes in protein abundances log-transformed iTRAQ ratios were analyzed using Student’s *t*-test and the threshold for differential abundance was p < 0.05. Gene Ontology analysis of proteins was carried out by using the PANTHER (Protein Analysis Through Evolutionary Relationships) classification system [99] and Swiss-Prot KB gene ontology data [100].

## Additional file

Additional file 1: Table S1. Proteins identified in human follicular fluid. Proteins detected in human follicular fluid. Proteins detected at 5% FDR in at least one sample were reported. The first column for each sample, FF1-FF3, “Rank” shows the ranking of the specified protein to all other proteins identified in that sample and the second column, “Unused ProtScor” is the ProteinPilot confidence score. Follicular fluid proteins also reported in Ambekar, et al., [21] and Twight et al., [31] are denoted by an “x”. Proteins detected for the first time in FF are in bold. Farrah et al., [34] shows FF proteins that are in the Human Plasma PeptideAtlas list and Balakrishnan et al., [39] shows proteins detected in synovial fluid. Follicular fluid proteins were compared to granulosa cell transcriptomic data from Koks et al., [64] and the corresponding FF proteins denoted with an “x”. Mural granulosa cells, mGC; cumulus granulosa cells, cGC.

## Abbreviations

ART: Assisted reproductive technology
FF: Human follicular fluid
IGF: Insulin growth factor
IVF: In vitro fertilization

## References

[1] Rodgers RJ, Irving-Rodgers HF (2010). Formation of the ovarian follicular antrum and follicular fluid. Biol Reprod.

[2] Shalgi R, Kraicer P, Rimon A, Pinto M, Soferman N (1973). Proteins of human follicular fluid: the blood-follicle barrier. Fertil Steril.

[3] Hess KA, Chen L, Larsen WJ (1998). The ovarian blood follicle barrier is both charge- and size-selective in mice. Biol Reprod.

[4] Park JY, Su YQ, Ariga M, Law E, Jin SL, Conti M (2004). EGF-like growth factors as mediators of LH action in the ovulatory follicle. Science.

[5] Russell DL, Robker RL (2007). Molecular mechanisms of ovulation: co-ordination through the cumulus complex. Hum Reprod Update.

[6] Zhang M, Su YQ, Sugiura K, Xia G, Eppig JJ (2010). Granulosa cell ligand NPPC and its receptor NPR2 maintain meiotic arrest in mouse oocytes. Science.

[7] Chang CL, Wang HS, Soong YK, Huang SY, Pai SY, Hsu SY (2011). Regulation of oocyte and cumulus cell interactions by intermedin/adrenomedullin 2. J Biol Chem.

[8] Gilchrist RB, Lane M, Thompson JG (2008). Oocyte-secreted factors: regulators of cumulus cell function and oocyte quality. Hum Reprod Update.

[9] Gui LM, Joyce IM (2005). RNA interference evidence that growth differentiation factor-9 mediates oocyte regulation of cumulus expansion in mice. Biol Reprod.

[10] Balasch J, Guimera M, Martinez-Pasarell O, Ros J, Vanrell JA, Jimenez W (2004). Adrenomedullin and vascular endothelial growth factor production by follicular fluid macrophages and granulosa cells. Hum Reprod.

[11] Becker S, von Otte S, Robenek H, Diedrich K, Nofer JR (2011). Follicular fluid high-density lipoprotein-associated sphingosine 1-phosphate (S1P) promotes human granulosa lutein cell migration via S1P receptor type 3 and small G-protein RAC1. Biol Reprod.

[12] Byskov AG, Andersen CY, Leonardsen L (2002). Role of meiosis activating sterols, MAS, in induced oocyte maturation. Mol Cell Endocrinol.

[13] Norris RP, Ratzan WJ, Freudzon M, Mehlmann LM, Krall J, Movsesian MA (2009). Cyclic GMP from the surrounding somatic cells regulates cyclic AMP and meiosis in the mouse oocyte. Development.

[14] Vaccari S, Weeks JL, Hsieh M, Menniti FS, Conti M (2009). Cyclic GMP signaling is involved in the luteinizing hormone-dependent meiotic maturation of mouse oocytes. Biol Reprod.

[15] Hillier SG (2009). Paracrine support of ovarian stimulation. Mol Hum Reprod.

[16] Hsieh M, Zamah AM, Conti M (2009). Epidermal growth factor-like growth factors in the follicular fluid: role in oocyte development and maturation. Semin Reprod Med.

[17] Knight PG, Satchell L, Glister C (2012). Intra-ovarian roles of activins and inhibins. Mol Cell Endocrinol.

[18] Mottershead DG, Ritter LJ, Gilchrist RB (2012). Signalling pathways mediating specific synergistic interactions between GDF9 and BMP15. Mol Hum Reprod.

[19] Otsuka F (2010). Multiple endocrine regulation by bone morphogenetic protein system. Endocr J.

[20] Angelucci S, Ciavardelli D, Di Giuseppe F, Eleuterio E, Sulpizio M, Tiboni GM (2006). Proteome analysis of human follicular fluid. Biochim Biophys Acta.

[21] Ambekar AS, Nirujogi RS, Srikanth SM, Chavan S, Kelkar DS, Hinduja I (2013). Proteomic analysis of human follicular fluid: a new perspective towards understanding folliculogenesis. J Proteomics.

[22] Estes SJ, Ye B, Qiu W, Cramer D, Hornstein MD, Missmer SA (2009). A proteomic analysis of IVF follicular fluid in women < or = 32 years old. Fertil Steril.

[23] Liu AX, Zhu YM, Luo Q, Wu YT, Gao HJ, Zhu XM (2007). Specific peptide patterns of follicular fluids at different growth stages analyzed by matrix-assisted laser desorption/ionization time-of-flight mass spectrometry. Biochim Biophys Acta.

[24] Hanrieder J, Nyakas A, Naessen T, Bergquist J (2008). Proteomic analysis of human follicular fluid using an alternative bottom-up approach. J Proteome Res.

[25] Hanrieder J, Zuberovic A, Bergquist J (2009). Surface modified capillary electrophoresis combined with in solution isoelectric focusing and MALDI-TOF/TOF MS: a gel-free multidimensional electrophoresis approach for proteomic profiling–exemplified on human follicular fluid. J Chromatogr A.

[26] Jarkovska K, Martinkova J, Liskova L, Halada P, Moos J, Rezabek K (2010). Proteome mining of human follicular fluid reveals a crucial role of complement cascade and key biological pathways in women undergoing in vitro fertilization. J Proteome Res.

[27] Kim YS, Kim MS, Lee SH, Choi BC, Lim JM, Cha KY (2006). Proteomic analysis of recurrent spontaneous abortion: Identification of an inadequately expressed set of proteins in human follicular fluid. Proteomics.

[28] Anderson NL, Anderson NG (2002). The human plasma proteome: history, character, and diagnostic prospects. Mol Cell Proteomics.

[29] Wen X, Perrett D, Patel P, Li N, Docherty SM, Tozer AJ (2009). Capillary electrophoresis of human follicular fluid. J Chromatogr B Analyt Technol Biomed Life Sci.

[30] Anahory T, Dechaud H, Bennes R, Marin P, Lamb NJ, Laoudj D (2002). Identification of new proteins in follicular fluid of mature human follicles. Electrophoresis.

[31] Twigt J, Steegers-Theunissen RP, Bezstarosti K, Demmers JA (2012). Proteomic analysis of the microenvironment of developing oocytes. Proteomics.

[32] Lygirou V, Makridakis M, Vlahou A (2015). Biological sample collection for clinical proteomics: existing SOPs. Methods Mol Biol.

[33] Schmidt A, Aebersold R (2006). High-accuracy proteome maps of human body fluids. Genome Biol.

[34] Farrah T, Deutsch EW, Omenn GS, Campbell DS, Sun Z, Bletz JA (2011). A high-confidence human plasma proteome reference set with estimated concentrations in PeptideAtlas. Mol Cell Proteomics.

[35] de Agostini AI, Dong JC, de Vantery AC, Ramus MA, Dentand-Quadri I, Thalmann S (2008). Human follicular fluid heparan sulfate contains abundant 3-O-sulfated chains with anticoagulant activity. J Biol Chem.

[36] Kamat BR, Brown LF, Manseau EJ, Senger DR, Dvorak HF (1995). Expression of vascular permeability factor/vascular endothelial growth factor by human granulosa and theca lutein cells role in corpus luteum development. Am J Pathol.

[37] Bungay SD, Gentry PA, Gentry RD (2006). Modelling thrombin generation in human ovarian follicular fluid. Bull Math Biol.

[38] Cheng Y, Kawamura K, Deguchi M, Takae S, Mulders SM, Hsueh AJ (2012). Intraovarian thrombin and activated protein C signaling system regulates steroidogenesis during the periovulatory period. Mol Endocrinol.

[39] Balakrishnan L, Bhattacharjee M, Ahmad S, Nirujogi RS, Renuse S, Subbannayya Y (2014). Differential proteomic analysis of synovial fluid from rheumatoid arthritis and osteoarthritis patients. Clin Proteomic.

[40] Hester KE, Harper MJ, Duffy DM (2010). Oral administration of the cyclooxygenase-2 (COX-2) inhibitor meloxicam blocks ovulation in non-human primates when administered to simulate emergency contraception. Hum Reprod.

[41] Jesam C, Salvatierra AM, Schwartz JL, Croxatto HB (2010). Suppression of follicular rupture with meloxicam, a cyclooxygenase-2 inhibitor: potential for emergency contraception. Hum Reprod.

[42] Fang Q, Kani K, Faca VM, Zhang W, Zhang Q, Jain A (2011). Impact of protein stability, cellular localization, and abundance on proteomic detection of tumor-derived proteins in plasma. PLoS One.

[43] Huntington JA (2011). Serpin structure, function and dysfunction. J Thromb Haemost.

[44] Ledee N, Lombroso R, Lombardelli L, Selva J, Dubanchet S, Chaouat G (2008). Cytokines and chemokines in follicular fluids and potential of the corresponding embryo: the role of granulocyte colony-stimulating factor. Hum Reprod.

[45] Vujisic S, Lepej SZ, Emedi I, Bauman R, Remenar A, Tiljak MK (2006). Ovarian follicular concentration of IL-12, IL-15, IL-18 and p40 subunit of IL-12 and IL-23. Hum Reprod.

[46] Bianchi L, Gagliardi A, Campanella G, Landi C, Capaldo A, Carleo A (2013). A methodological and functional proteomic approach of human follicular fluid en route for oocyte quality evaluation. J Proteomic.

[47] Conti M, Hsieh M, Zamah AM, Oh JS (2012). Novel signaling mechanisms in the ovary during oocyte maturation and ovulation. Mol Cell Endocrinol.

[48] Sobinoff AP, Sutherland JM, McLaughlin EA (2013). Intracellular signalling during female gametogenesis. Mol Hum Reprod.

[49] Kwintkiewicz J, Giudice LC (2009). The interplay of insulin-like growth factors, gonadotropins, and endocrine disruptors in ovarian follicular development and function. Semin Reprod Med.

[50] Horka P, Malickova K, Jarosova R, Janatkova I, Zima T, Kalousova M (2012). Matrix metalloproteinases in serum and the follicular fluid of women treated by in vitro fertilization. J Assist Reprod Genet.

[51] Hayashi KG, Ushizawa K, Hosoe M, Takahashi T (2010). Differential genome-wide gene expression profiling of bovine largest and second-largest follicles: identification of genes associated with growth of dominant follicles. Reprod Biol Endocrinol.

[52] Shalev E, Goldman S, Ben-Shlomo I (2001). The balance between MMP-9 and MMP-2 and their tissue inhibitor (TIMP)-1 in luteinized granulosa cells: comparison between women with PCOS and normal ovulatory women. Mol Hum Reprod.

[53] Stilley JA, Sharpe-Timms KL (2012). TIMP1 contributes to ovarian anomalies in both an MMP-dependent and -independent manner in a rat model. Biol Reprod.

[54] Ruvolo G, Fattouh RR, Bosco L, Brucculeri AM, Cittadini E (2013). New molecular markers for the evaluation of gamete quality. J Assist Reprod Genet.

[55] Mellgren RL (2010). A plasma membrane wound proteome: reversible externalization of intracellular proteins following reparable mechanical damage. J Biol Chem.

[56] Schenk S, Schoenhals GJ, de Souza G, Mann M (2008). A high confidence, manually validated human blood plasma protein reference set. BMC Med Genomics.

[57] Russell DL, Salustri A (2006). Extracellular matrix of the cumulus-oocyte complex. Semin Reprod Med.

[58] Lee HC, Lee SW, Lee KW, Lee SW, Cha KY, Kim KH (2005). Identification of new proteins in follicular fluid from mature human follicles by direct sample rehydration method of two-dimensional polyacrylamide gel electrophoresis. J Korean Med Sci.

[59] Anderson L, Hunter CL (2006). Quantitative mass spectrometric multiple reaction monitoring assays for major plasma proteins. Mol Cell Proteomics.

[60] Bisson N, James DA, Ivosev G, Tate SA, Bonner R, Taylor L (2011). Selected reaction monitoring mass spectrometry reveals the dynamics of signaling through the GRB2 adaptor. Nat Biotechnol.

[61] Razavi M, Frick LE, LaMarr WA, Pope ME, Miller CA, Anderson NL (2012). High-throughput SISCAPA quantitation of peptides from human plasma digests by ultrafast, liquid chromatography-free mass spectrometry. J Proteome Res.

[62] Joseph C, Hunter MG, Sinclair KD, Robinson RS (2012). The expression, regulation and function of secreted protein, acidic, cysteine-rich in the follicle-luteal transition. Reproduction.

[63] Rasanen J, Girsen A, Lu X, Lapidus JA, Standley M, Reddy A (2010). Comprehensive maternal serum proteomic profiles of preclinical and clinical preeclampsia. J Proteome Res.

[64] Koks S, Velthut A, Sarapik A, Altmae S, Reinmaa E, Schalkwyk LC (2010). The differential transcriptome and ontology profiles of floating and cumulus granulosa cells in stimulated human antral follicles. Mol Hum Reprod.

[65] Gosden RG (2002). Oogenesis as a foundation for embryogenesis. Mol Cell Endocrinol.

[66] Zuccotti M, Merico V, Cecconi S, Redi CA, Garagna S (2011). What does it take to make a developmentally competent mammalian egg?. Hum Reprod Update.

[67] Akande AV, Asselin J, Keay SD, Cahill DJ, Muttukrishna S, Groome NP (2000). Inhibin A, inhibin B and activin A in follicular fluid of infertile women with tubal damage, unexplained infertility and emdometriosis. Am J Reprod Immunol.

[68] Gentry PA, Plante L, Schroeder MO, LaMarre J, Young JE, Dodds WG (2000). Human ovarian follicular fluid has functional systems for the generation and modulation of thrombin. Fertil Steril.

[69] Jarkovska K, Kupcova Skalnikova H, Halada P, Hrabakova R, Moos J, Rezabek K (2011). Development of ovarian hyperstimulation syndrome: interrogation of key proteins and biological processes in human follicular fluid of women undergoing in vitro fertilization. Mol Hum Reprod.

[70] Kleinman D, Insler V, Leiberman JR, Glezerman M, Albotiano S, Potashnik G (1987). Acid phosphatase levels in follicular fluids following induction of ovulation in in vitro fertilization patients. J In Vitro Fert Embryo Transf.

[71] Nordqvist S, Karehed K, Hambiliki F, Wanggren K, Stavreus-Evers A, Akerud H (2010). The presence of histidine-rich glycoprotein in the female reproductive tract and in embryos. Reprod Sci.

[72] Rodgers RJ, Irving-Rodgers HF (2010). The roles of the ovarian extracellular matrix in fertility. Soc Reprod Fertil Suppl.

[73] Singh AK, Chattopadhyay R, Chakravarty B, Chaudhury K (2013). Altered circulating levels of matrix metalloproteinases 2 and 9 and their inhibitors and effect of progesterone supplementation in women with endometriosis undergoing in vitro fertilization. Fertil Steril.

[74] Yoo SW, Bolbot T, Koulova A, Sneeringer R, Humm K, Dagon Y (2013). Complement factors are secreted in human follicular fluid by granulosa cells and are possible oocyte maturation factors. J Obstet Gynaecol Res.

[75] Goldman S, Shalev E (2004). MMPS and TIMPS in ovarian physiology and pathophysiology. Front Biosci.

[76] Roberts VJ, Barth S, El-Roeiy A, Yen SS (1993). Expression of inhibin/activin subunits and follistatin messenger ribonucleic acids and proteins in ovarian follicles and the corpus luteum during the human menstrual cycle. J Clin Endocrinol Metab.

[77] Babayof R, Margalioth EJ, Huleihel M, Amash A, Zylber-Haran E, Gal M (2006). Serum inhibin A, VEGF and TNFalpha levels after triggering oocyte maturation with GnRH agonist compared with HCG in women with polycystic ovaries undergoing IVF treatment: a prospective randomized trial. Hum Reprod.

[78] Suresh PS, Medhamurthy R (2012). Luteinizing hormone regulates inhibin-alpha subunit expression through multiple signaling pathways involving steroidogenic factor-1 and beta-catenin in the macaque corpus luteum. Growth Factors.

[79] Fujimoto N, Terlizzi J, Aho S, Brittingham R, Fertala A, Oyama N (2006). Extracellular matrix protein 1 inhibits the activity of matrix metalloproteinase 9 through high-affinity protein/protein interactions. Exp Dermatol.

[80] Poon IK, Patel KK, Davis DS, Parish CR, Hulett MD (2011). Histidine-rich glycoprotein: the Swiss Army knife of mammalian plasma. Blood.

[81] Gorgani NN, Parish CR, Easterbrook Smith SB, Altin JG (1997). Histidine-rich glycoprotein binds to human IgG and C1q and inhibits the formation of insoluble immune complexes. Biochemistry.

[82] Lijnen HR, Hoylaerts M, Collen D (1983). Heparin binding properties of human histidine-rich glycoprotein mechanism and role in the neutralization of heparin in plasma. J Biol Chem.

[83] Simantov R, Febbraio M, Crombie R, Asch AS, Nachman RL, Silverstein RL (2001). Histidine-rich glycoprotein inhibits the antiangiogenic effect of thrombospondin-1. J Clin Invest.

[84] Jones AL, Hulett MD, Parish CR (2005). Histidine-rich glycoprotein: a novel adaptor protein in plasma that modulates the immune, vascular and coagulation systems. Immunol Cell Biol.

[85] Lindgren KE, Karehed K, Karypidis H, Hosseini F, Bremme K, Landgren BM (2013). Histidine-rich glycoprotein gene polymorphism in patients with recurrent miscarriage. Acta Obstet Gynecol Scand.

[86] Nordqvist S, Karehed K, Stavreus-Evers A, Akerud H (2011). Histidine-rich glycoprotein polymorphism and pregnancy outcome: a pilot study. Reprod Biomed Online.

[87] Artini PG, Monti M, Matteucci C, Valentino V, Cristello F, Genazzani AR (2006). Vascular endothelial growth factor and basic fibroblast growth factor in polycystic ovary syndrome during controlled ovarian hyperstimulation. Gynecol Endocrinol.

[88] Soares SR (2012). Etiology of OHSS and use of dopamine agonists. Fertil Steril.

[89] Van Blerkom J, Antczak M, Schrader R (1997). The developmental potential of the human oocyte is related to the dissolved oxygen content of follicular fluid: association with vascular endothelial growth factor levels and perifollicular blood flow characteristics. Hum Reprod.

[90] Dixelius J, Olsson AK, Thulin A, Lee C, Johansson I, Claesson-Welsh L (2006). Minimal active domain and mechanism of action of the angiogenesis inhibitor histidine-rich glycoprotein. Cancer Res.

[91] Lee A, Christenson LK, Stouffer RL, Burry KA, Patton PE (1997). Vascular endothelial growth factor levels in serum and follicular fluid of patients undergoing in vitro fertilization. Fertil Steril.

[92] Severino V, Malorni L, Cicatiello AE, D'Esposito V, Longobardi S, Colacurci N (2013). An integrated approach based on multiplexed protein array and iTRAQ labeling for in-depth identification of pathways associated to IVF outcome. PLoS One.

[93] Zamah AM, Hsieh M, Chen J, Vigne JL, Rosen MP, Cedars MI (2010). Human oocyte maturation is dependent on LH-stimulated accumulation of the epidermal growth factor-like growth factor, amphiregulin. Hum Reprod.

[94] Anderson RA, Sciorio R, Kinnell H, Bayne RA, Thong KJ, de Sousa PA (2009). Cumulus gene expression as a predictor of human oocyte fertilisation, embryo development and competence to establish a pregnancy. Reproduction.

[95] Fragouli E, Wells D, Iager AE, Kayisli UA, Patrizio P (2012). Alteration of gene expression in human cumulus cells as a potential indicator of oocyte aneuploidy. Hum Reprod.

[96] Wathlet S, Adriaenssens T, Segers I, Verheyen G, Janssens R, Coucke W (2012). New candidate genes to predict pregnancy outcome in single embryo transfer cycles when using cumulus cell gene expression. Fertil Steril.

[97] Rosen MP, Zamah AM, Shen S, Dobson AT, McCulloch CE, Rinaudo PF (2009). The effect of follicular fluid hormones on oocyte recovery after ovarian stimulation: FSH level predicts oocyte recovery. Reprod Biol Endocrinol.

[98] Tang WH, Shilov IV, Seymour SL (2008). Nonlinear fitting method for determining local false discovery rates from decoy database searches. J Proteome Res.

[99] Thomas PD, Campbell MJ, Kejariwal A, Mi H, Karlak B, Daverman R (2003). PANTHER: a library of protein families and subfamilies indexed by function. Genome Res.

[100] Fernandez-Suarez XM, Galperin MY (2013). The 2013 nucleic acids research database issue and the online molecular biology database collection. Nucleic Acids Res.

